# The oncogene Gankyrin is expressed in testicular cancer and contributes to cisplatin sensitivity in embryonal carcinoma cells

**DOI:** 10.1186/s12885-019-6340-7

**Published:** 2019-11-19

**Authors:** Maria E. Camacho-Moll, Joni Macdonald, L. H. J. Looijenga, Michael P. Rimmer, Roland Donat, John A. Marwick, C. J. Shukla, Neil Carragher, Anne Jørgensen, Rod T. Mitchell

**Affiliations:** 1 Departamento de Biología Molecular, Centro de Investigación Biomédica del Noreste, Delegación Nuevo León, Instituto Mexicano del Seguro Social, Calle 2 de abril 501, esq. San Luis Potosí, Col. Independencia, CP, 64720 Monterrey, Nuevo León Mexico; 2 Centro de Diagnóstico Molecular y Medicina Personalizada, División Ciencias de la Salud, Universidad de Monterrey, Av. Ignacio Morones Prieto 4500 Pte, N. L, 66238 San Pedro Garza García, Mexico; 3 MRC Centre for Reproductive Health, The University of Edinburgh, Queen’s Medical Research Institute, 47 Little France Crescent, Edinburgh, Scotland, EH16 4TJ UK; 4 Department of Pathology, Erasmus University, Medical Center, Cancer Center, Josephine Nefkens Institute, Wytemaweg 80, 3015 Rotterdam, CN Netherlands; 5 Princess Maxima Center for Pediatric Oncology, Heidelberglaan 25, 3584 CS Utrecht, The Netherlands; 6 Department of Urology, Western General Hospital, Crewe Road, Edinburgh, Scotland, EH4 2XU UK; 7 The MRC Centre for Inflammation Research, Queen’s Medical Research Institute, University of Edinburgh, 47 Little France Crescent, Edinburgh, EH16 4TJ UK; 8 Cancer Research UK Edinburgh Centre, MRC Institute of Genetics and Molecular Medicine, University of Edinburgh, Edinburgh, UK; 9 Department of Growth and Reproduction, University Hospital of Copenhagen, Rigshospitalet, Blegdamsvej 9 2100 KBH Ø, Copenhagen, UK

**Keywords:** Gankyrin, Testicular germ cell cancer, GCNIS, Apoptosis, Cisplatin sensitivity

## Abstract

**Background:**

Testicular germ cell cancer (TGCC) develops from pre-malignant germ neoplasia in situ (GCNIS) cells. GCNIS originates from fetal gonocytes (POU5F1^+^/MAGE-A4^−^), which fail to differentiate to pre-spermatogonia (POU5F1^−^/MAGE-A4^+^) and undergo malignant transformation. Gankyrin is an oncogene which has been shown to prevent POU5F1 degradation and specifically interact with MAGE-A4 in hepatocellular carcinoma (HCC) cells. We aimed to investigate the role of Gankyrin in progression from gonocyte to pre-invasive GCNIS and subsequent invasive TGCC.

**Methods:**

We determined Gankyrin expression in human fetal testicular tissue (gestational weeks 9–20; *n* = 38), human adult testicular tissue with active spermatogenesis (*n* = 9), human testicular tissue with germ cell maturation delay (*n* = 4), testicular tissue from patients with pre-invasive GCNIS (*n* = 6), and invasive TGCC including seminoma (n = 6) and teratoma (*n* = 7). Functional analysis was performed in-vitro by siRNA knock-down of Gankyrin in the NTera2 cells (derived from embryonal carcinoma).

**Results:**

Germ cell expression of Gankyrin was restricted to a sub-population of prespermatogonia in human fetal testes. Nuclear Gankyrin was also expressed in GCNIS cells of childhood and adult pre-invasive TGCC patients, and in GCNIS from seminoma and non-seminoma patients. Cytoplasmic expression was observed in seminoma tumour cells and NTera2 cells. Gankyrin knock-down in NTera2 cells resulted in an increase in apoptosis mediated via the TP53 pathway, whilst POU5F1 expression was unaffected. Furthermore, Gankyrin knock-down in NTera2 cells increased cisplatin sensitivity with an increase in cell death (13%, *p* < 0.05) following Gankyrin knock-down, when compared to cisplatin treatment alone, likely via BAX and FAS. Our results demonstrate that Gankyrin expression changes in germ cells during normal transition from gonocyte to prespermatogonia. In addition, changes in Gankyrin localisation are associated with progression of pre-invasive GCNIS to invasive TGCC. Furthermore, we found that Gankyrin is involved in the regulation of NTera2 cell survival and that a reduction in Gankyrin expression can modulate cisplatin sensitivity.

**Conclusions:**

These results suggest that manipulation of Gankyrin expression may reduce the cisplatin dose required for the treatment of TGCC, with benefits in reducing dose-dependent side effects of chemotherapy. Further studies are required in order to assess the effects of modulating Gankyrin on GCNIS/TGCC using in vivo models.

## Background

The incidence of testicular germ cell cancer (TGCC) has increased over recent decades and is currently the most common malignancy among young caucasian men [1–3]. The precursor lesion for TGCC, known as Germ Cell Neoplasia in situ (GCNIS, previously carcinoma in situ) originates during fetal life when a sub-population of gonocytes fail to differentiate normally to (pre) spermatogonia [4, 5]. Several studies have shown that GCNIS cells resemble gonocytes with regard to morphology, epigenetic profile and protein expression [6–8]. GCNIS cells remain dormant in the testis until after puberty, when they gain invasive capacity and develop into invasive tumours [9, 10], histologically classified as seminoma and non-seminoma [11]. Although models for the pathogenesis of TGCC have been hypothesized [12], the mechanisms that result in failure of gonocyte differentiation, development of GCNIS, and stimulation of proliferation of GCNIS to gain invasive capacity to form TGCC are incompletely understood, although an interaction within (epi) genetics and environmental parameters are assumed [13].

All gonocytes and GCNIS cells express the pluripotency factor POU5F1 (OCT4), whilst a sub-population of GCNIS cells also express the (pre) spermatogonial protein MAGE-A4. We have previously shown that MAGE-A4^−^ GCNIS cells proliferate more frequently than the MAGE-A4^+^ population, suggesting that MAGE-A4 might have an anti-proliferative effect when expressed in GCNIS cells [14].

Gankyrin is an oncogene that has previously been shown to be involved in the pathogenesis of several cancers including colorectal cancer [15, 16], breast cancer [17–19] and hepatocelullar carcinoma [20–25]. MAGE-A4 has been shown to suppress the oncogenic properties of Gankyrin leading to reduced tumour size in a mouse model of Gankyrin overexpression [26]. Gankyrin also prevents POU5F1 degradation in hepatocellular carcinoma (HCC) by inhibiting the interaction of POU5F1 with WW domain containing E3 ubiquitin protein ligase (WWP2 [23, 27];. POU5F1 has also been shown to be negatively regulated by WWP2 in NTera2 (embryonal carcinoma) cells [27].

Cisplatin based chemotherapy is frequently used for treatment of TGCC, resulting in a high cure rate [28, 29]. However, cisplatin treatment also results in significant adverse effects in patients which includes infertility. It has been hypothesized that the efficiency of cisplatin based chemotherapy in TGCC is due at least in part to the high expression of wildtype TP53 in TGCC [30, 31]. It has been previously shown that TP53 is required by cisplatin to facilitate cytotoxicity [32] and that the predominant mechanism of cisplatin cytotoxicity in TGCC is due toTP53 hypersensitivity [33]. This has been demonstrated in the TGCC cell line NTera2 and 2102EP [34]. Gankyrin has been linked to TP53 in HCC [22, 35], where it has been shown that Gankyrin mediates the degradation of tumour suppressor proteins such as TP53 [36, 37]. However, there have been no previous studies examining the effect of Gankyrin manipulation on TP53 and downstream signalling in TGCC.

We hypothesized that Gankyrin expression in the human fetal testis is associated with germ cell maturation and malignant transformation. We also hypothesized that Gankyrin regulates the oncogenic potential and cisplatin sensitivity of NTera2 cells via the TP53 pathway.

## Methods

The study aim was to characterize Gankyrin expression in human testicular tissues from fetal life through adulthood and to compare with GCNIS and TGCC. Furthermore, we aimed to investigate the impact of Gankyrin knockdown on cell death and cisplatin sensitivity in embryonal carcinoma cells.

### Tissue collection

#### Human fetal testis tissue

Human fetal testicular tissue was obtained following elective termination of pregnancy during gestational weeks 9 to 20 (*n* = 38). Women gave informed consent and tissue was collected with ethical approval (REC reference: LREC08/1101/1 and 08/H0906/21 + 5). Gestational age was determined by ultrasound and confirmed by measuring foot length. Testis tissue was fixed in formalin for 24 h, transferred into 70% ethanol and then embedded in paraffin. Sections of 5 μm thickness were prepared. Sex was determined by expression of sex determining region gene Y (SRY) gene by qPCR as previously described [38].

#### Adult testicular tissue

Testicular tissue with complete spermatogenesis (*n* = 9) was obtained from archived material. Orchiectomy was performed for clinical indications primarily involving chronic testicular pain. Tissues were fixed in buffered formalin for pathological assessment. Ethical approval was obtained for the use of archived human testicular tissue from the pathology department at the Western General Hospital in Edinburgh (REC Reference: 10/S1402/33) and from the biobank at the Department of Growth and Reproduction, University Hospital of Copenhagen, Denmark (H-1-2012-007).

#### Childhood testicular samples with maturation delay

Tissue from children aged 0–2 years with maturation delay (*n* = 4) were obtained from Erasmus MC-University Medical Center, Rotterdam (Institutional review board – MEC 02.981 and CCR2041). Tissues were obtained in the context of routine clinical diagnosis of suspected gonadal anomalies to be evaluated by histological examination. The samples were handled according to existing standard operational protocols and evaluated by an experienced clinical uro-pathologist. Left over tissue was approved to be used for scientific research. The diagnosis of maturation delay is reached when in children above 6 months, POU5F1^+^ germ cells with round nuclei present in the lumen of a seminiferous tubule can be observed [39].

#### TGCC samples

Tissue from children (*n* = 2) and adults (n = 4) with pre-invasive disease (pre-GCNIS cells or GCNIS cells without evidence of invasive tumour) were obtained from Erasmus MC-University Medical Center, Rotterdam (Institutional review board – MEC 02.981 and CCR2041) and from the biobank at the Department of Growth and Reproduction, University Hospital of Copenhagen, Copenhagen, Denmark (H-1-2012-007). Samples from pre-GCNIS patients were diagnosed by the presence of OCT4^+^ cells which can co-express TSPY in a heterogeneous pattern accompanied by focal KITLG expression. These cells been relocated from the center of the tubule to the pre-spermatogonial niche at the basement membrane [39]. These tissues were obtained from patients with Disorders of Sexual Development (DSD), infertility, or suspected TGCC for diagnostic purposes. Invasive TGCC tissue was obtained from clinical orchiectomy specimens from men with seminoma, (*n* = 6) and teratoma (*n* = 7). These tissues contained regions with histologically normal spermatogenesis, GCNIS cells or the tumour component. Tissues were randomly selected and the presence of GCNIS cells was confirmed by light microscopy prior to commencing the study.

### Triple immunofluorescence

Sections were dewaxed in xylene, rehydrated in graded alcohols and washed in tap water. Antigen retrieval was performed in 0.01 M citrate buffer in a decloaking chamber (Biocare Medical, Berkshire, UK), sections were then washed in tap water and endogenous peroxidase was blocked with 3% H_2_O_2_ in MeOH for 30 min, followed by two washes in Tris Buffer saline (TBS) for 5 min. Sections were blocked with Normal chicken serum (NChS) for 30 min at room temperature (RT) followed by incubation with POU5F1 antibody (Santa Cruz, Heidelberg, Germany – sc8628) overnight at 4 °C. The following day sections were washed twice with TBS for 5 min each and incubated with chicken anti goat peroxidase labelled antibody (1 in 200 in NChS) for 30 min, followed by two 5 min washes with TBS and incubation with Tyramide signal amplification (TSA, Perkin Elmer, Waltham, USA), at 1:50 for 10 min. Sections were microwaved for 2.5 min in 0.01 M citrate buffer, followed by a 30 min cool down period. The process from NChS block up to primary antibody detection was repeated twice more for two subsequent primary antibodies MAGE-A4 (gift, described in [40, 41]) and Gankyrin (Novus Bio – NBP1–82443). Sections were counterstained with DAPI (4′, 6-diamidino-2-phenylindole, Sigma, Poole, UK) by adding 1 μl/mL of TBS and incubating the sections for 10 min in the dark. Finally, sections were washed twice with TBS for 5 min and mounted with PermaFluor (Life Technologies, Paisley, UK). Negative controls were no primary antibody controls and are shown as insets. Details of antibodies used for immunofluorescence can be found in Table 1.

**Table 1:** Details of primary antibodies

Antibody	Laboratory	Immunofluorescence	Western blot
Gankyrin	Novus	1 in 10,000	1 in 1000
GAPDH	Abcam	N/A	1:5000
MAGE-A4	gift	1:300	N/A
POU5F1	Santa Cruz	1:150	1:300
SOX9	Merck Millipore	1:5000	N/A
TP53	Santa Cruz	1:1000	1:1000
Tubulin	Abcam	N/A	1:5000

### Western blot

NTera2 cells were resuspended in 50 μl of radioimmunoprecipitation assay (RIPA) buffer with protease inhibitors (Roche, Baser, Switzerland). Bradford assay was used to quantify the amount of protein in each sample and Western blot was performed with 20 μg of protein in each lane. Protein was loaded into wells of a NOVEX SDS/PAGE (Life Technologies) gel, which was run at 150 mA with running buffer (Thermofisher scientific). Protein was transferred to an Amersham Hybond ECL nitrocellulose membrane (GE Healthcare Lifesciences, Buckinghamshire, UK) at 400 V, 250 mA and 50 W for 90 min. Membranes were then blocked with 5% skimmed milk powder in PBST (PBS + Tween®20) for 30 min at RT, followed by incubation overnight with the relevant primary antibody diluted in 5% skimmed milk in PBST at 4 °C. The following day membranes were incubated for 30 min at RT with a secondary antibody conjugated with IRDye 680 or 800 (LI-COR Biosciences, Nebraska, USA) at a concentration of 1 in 10,000, and scanned in the LI-COR Odyssey scanner (LI-COR Biosciences). Images were captured by Image Studio™ (Li-COR Biosciences) software. Tubulin or Glyceraldehyde 3-phosphate dehydrogenase (GAPDH) detection were used as loading controls. Relative protein expression was quantified with Image Studio™_._. Primary antibody details can be found on Table 1.

### Quantification of nuclear Gankyrin expression

POU5F1 was used to detect GCNIS cells in triple immunostained sections from pre-invasive and invasive TGCC patients. For each section,10 random fields with GCNIS were quantified using a LSM710 Zeiss confocal microscope (Carl Zeiss). For sections containing smaller areas of GCNIS, all GCNIS cells were counted. These images were then compiled using Image J software (Image J, U. S. National Institutes of Health, Bethesda, Maryland, USA). POU5F1^+^/MAGE-A4^−^ and POU5F1^+^/MAGE-A4^−^ GCNIS cells were counted using the cell counter plug-in in the Image J software. Data was analysed with GraphPad Prism 6.04 (GraphPad software INC., La Jolla, USA).

### Gankyrin SiRNA transfection of NTera2 cells

NTera2 cells were cultured with DMEM +1x glutamine +1x penicillin/streptomycin +10x FCS (all reagents from ThermoFisher Scientific) at 37 °C and 5%CO_2_. Cells were seeded in 12 well plates with 10 × 10^4^ cells per well on the day prior to transfection. Cells were transfected with 20 nM of Gankyrin siRNA using HiPerFect (Qiagen, Redwood City, C. A, U.S.A) transfection reagent diluted in DMEM + glutamine. Gankyrin siRNA (Life Technologies, Paisley, UK) with the sequence 5′-UUU CGA AGC UGC AUA AUG UAA GGG A-3′ was used for transfection of NTera2 cells. Controls included a media only and siRNA control. After randomization of the plate, cells were incubated at 37 °C and 5% CO_2_ for 10 h with Gankyrin siRNA or siRNA control. After 10 h the media was discarded and replaced by pre-warmed complete medium (DMEM + 5%FCS + 1x penicillin/streptomycin +1x glutamine). Cells were then harvested 24 or 48 h after transfection had commenced. RNA or protein was extracted and stored at − 80 °C until further analysis. Cells were counted using the NucleoCounter NC-100 automated cell count system (Chemometec, Allerod, Denmark). For in vitro experiments, a minimum of three replicates per experiment were included. These three replicates were considered as the experimental unit (n). Experiments were repeated a minimum of three times (*n* = 3).

### Modulation of Cisplatin effects in NTera2 cells

#### Cisplatin kill curve in NTera2 cells

In order to determine the optimal cisplatin concentration for moderate (~ 50%) cell death, 12 well plates were seeded with 10 × 10^4^ NTera2 cells. The following day (D1), plates were randomized for treatment with 0.25, 0.5, 1, 2, 4, 8, 16 or 100 μM cisplatin for 24 h. On D2, media was discarded, cells were washed with 1 ml DPBS (GIBCO, Hemel Hempstead, UK), and cells were dissociated with 250 μl TrypLE™ Express (Life technologies) for 5 min at 37 °C. Pre-warmed complete medium (750 μl) was added and the suspension was collected. Cells were centrifuged for 5 min at 4000 rpm. Media was discarded, and cells resuspended in 1 ml media. From this suspension 100 μL was taken for cells counts in the NucleoCounter NC-100 Automated cell count system (Chemometec). The dose to be used in further experiments was 4 μM of cisplatin for 24 h which corresponded to ~ 50% cell death.

#### Gankyrin siRNA in cisplatin treated NTera2 cells

To investigate the effects of Gankyrin knock-down on cisplatin sensitivity on NTera2 cells, cisplatin treatments (24 h) was initiated following transfection with siRNA targeting Gankyrin expression for 24 h. Cells were then harvested and quantified in the NucleoCounter NC-100 automated cell count system (Chemometec).

### Cell cycle analysis

Cell cycle analysis was performed in a 5 laser LSR Fortessa (BD Bioscience) flow cytometer. DNA was stained with HOECHST (Cell Signalling) and viability assessment was performed with propidium iodide staining. After harvesting cells as described above for the modulation of cisplatin effects in NTera2 cells section, cells were resuspended in a 15 μg/μl HOECHST (diluted in 2%FCS DPBS) solution and incubated in the dark for 30 min at 37 °C prior to cell cycle analysis.

### Apoptosis assay

NTera2 cells were cultured with DMEM +1x glutamine + 10% FCS (ThermoFisher Scientific) at 37 °C and 5% CO2 using the IncuCyte Live Cell Analysis System (Sartorious, Goettingen, Germany). Cells were seeded in 96 well plates (Corning), 15 × 10^4^ cells per well 24 h prior transfection to reach 80–90% confluence. NTera2 cells were cultured with media only, Gankyrin siRNA (100 μM; Qiagen) or siRNA control. Staurosporine (300 nM; Sigma, Missouri, US) was used as a positive control for apoptosis. Following 10 h incubation, media was replaced with the fluorogenic caspase biosensor, NucView 488 (Biotium, Fremont, C. A, U.S.A) diluted in DMEM +1x glutamine + 10% FCS, to detect Cleaved Caspase-3 substrate in cells. Cells were visualised and images were obtained at 72 h, using an IncuCyte™ live cell imaging instrument and 20X Objective (Sartorius AG).

### qPCR

RNA was extracted as per manufacturer’s instructions using the Qiagen minikit (Qiagen, Hilden Germany), and RNA quality/quantity was assessed using a NanoDrop (Thermofisher Scientific, Massachusetts, US). cDNA synthesis was performed according to manufacturer’s instructions using the MAXIMA first strand cDNA synthesis kit (ThermoFisher Scientific). qPCR was performed with SYBR green (Agilent technologies, Santa Clara, California, USA), according to manufacturer’s instructions. *RPS20* was used as housekeeping gene [42] and expression levels of Gankyrin, *POU5F1*, *RB1*, *CDK4*; *TP53* and its downstream genes *P21*, *BAX*, *FAS*, *PAI*, *BAI* and proliferation genes *Ki67*, *PCNA* and *TPX2* was investigated. Primer details are described in Table 2.

**Table 2:** Primers sequences used for qPCR

Gene	Forward Primer	Reverse Primer
*BAI1*	CTAAGATGGCGAAGGTGGAG	CTGTGGGATGAGACGGATGT
*BAX*	GAGGATGATTGCCGCCGTGGACA	GGTGGGGGAGGAGGCTTGAGG
*FAS*	TGCACCCGGACCCAGAATAC	GAAGACAAAGCCACCCCAAGTTAG
*Gankyrin*	ATGAGGCTACAGCAATGCAC	TACTTGCTCCTTGGGACACC
*KI67*	GAGGTGTGCAGAAAATCCAAA	CTGTCCCTATGACTTCTGGTTGT
*P21*	CTGGCTCTTGATACCCCCCT	TCAACACTGAGACGGGCTCC
*PAI1*	TGCTGGTGAATGCCCTCTACT	CGGTCATTCCCAGGTTCTCTA
*PCNA*	ACACGATTGGCCCTAAAGTCTTCCC	GACCACTCTGCTACGCCTGCAACCG
*POU5F1*	CTTCTGCTTCAGGAGCTTGG	GAAGGAGAAGCTGGAGCAAA
*RPS20*	AACAAGCCGCAACGTAAAATC	ACGATCCCACGTCTTAGAACC
*SRY*	ACAGTAAAGGCAACGTCCAG	ATCTGCGGGAAGCAAACTGC
*TP53*	TCCACTACAACTACATGTGTAAC	GTGAAATATTCTCCATCCAGTG

### Statistical analysis

Statistical analysis for all experiments was performed by paired t-test using Graphpad prism 6.04 (GraphPad software INC., La Jolla, USA). For each experiment a minimum of n = 3 (each with at least 3 technical replicates) were used.

## Results

### Gankyrin is not expressed in gonocytes, but in a subset of pre-spermatogonia in normal human fetal and adult testis

**Fig. 1:**
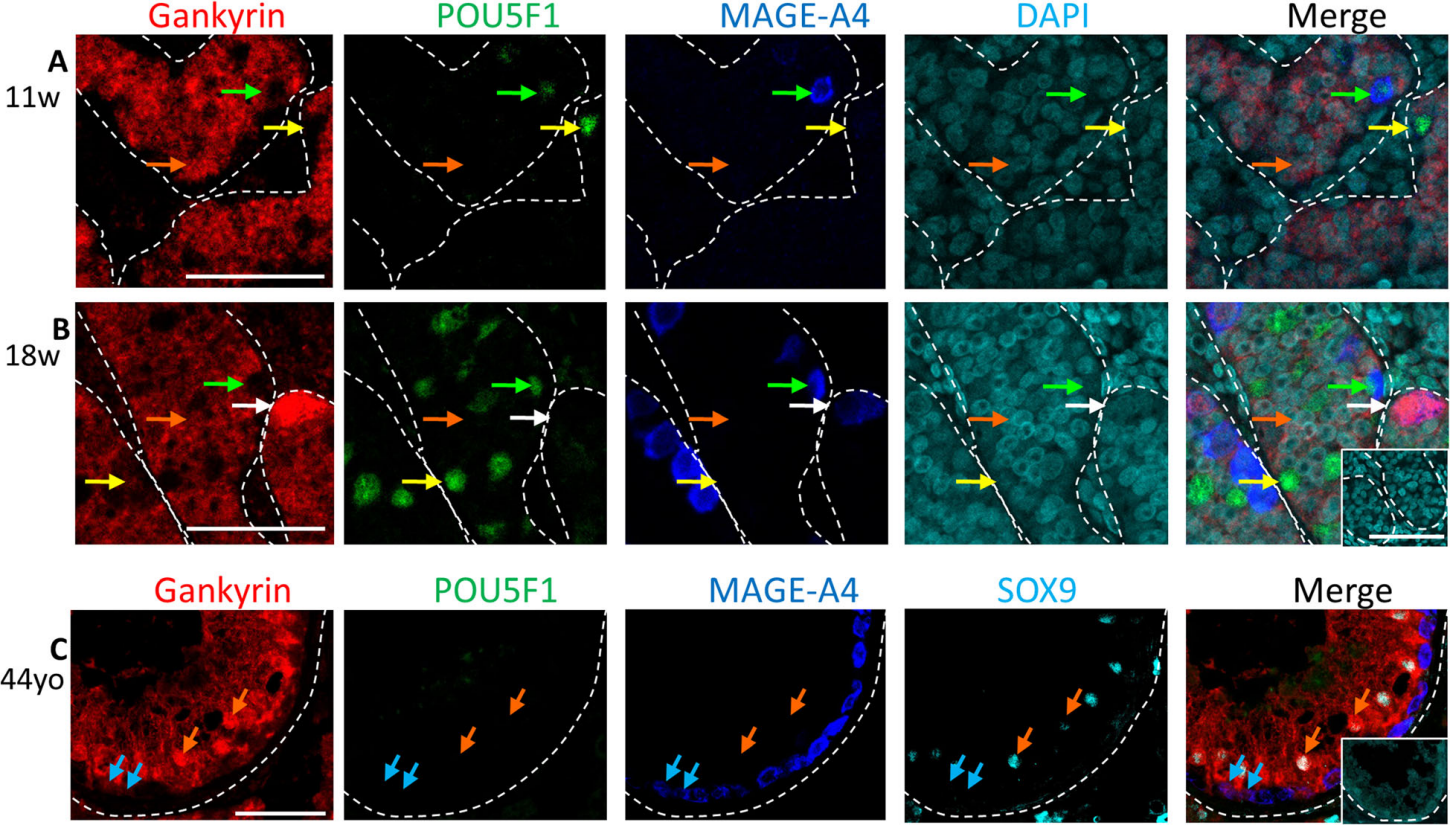
Representative images for Gankyrin expression in human fetal testis and normal adult testis. Gankyrin (red), POU5F1 (green; gonocyte) and MAGE-A4 (blue; prespermatogonia) expression in A), 11 week and B) 18 week old human fetal testis, counterstained with DAPI, yellow arrows – gonocytes, white arrows – pre-spermatogonia, green arrows – differentiating gonocyte, orange arrows – Sertoli cells, Scalebars – 50 μm. C) Representative image of Gankyrin expression in normal human adult testis immunostained for SOX9 (cyan; Sertoli cells), POU5F1 (green), MAGE-A4 (blue; spermatogonia) and Gankyrin (red), Cyan arrow – spermatogonia. Scalebars – 50 μm, insets – no primary antibody controls. w-weeks.

In human fetal testis, triple immunofluorescent staining with POU5F1, MAGE-A4 and Gankyrin was performed. Nuclear expression of Gankyrin could not be detected in gonocytes (POU5F1^+^/MAGE-A4^−^, yellow arrows, Fig. a and b), but in a subset of pre-spermatogonia (POU5F1^−^/MAGE-A4^+^, white arrows, Fig. 1b), nuclear Gankyrin expression was found. Gankyrin was also expressed in a subset of spermatogonia in adult testis tissue with full spermatogenesis (blue arrows, Fig. 1c). Abundant Sertoli cell expression of Gankyrin was present in both normal fetal and adult testis (orange arrows, Fig. 1).

### Nuclear Gankyrin is expressed in a subset of GCNIS in tissue from patients with pre-invasive or invasive TGCC

**Fig. 2:**
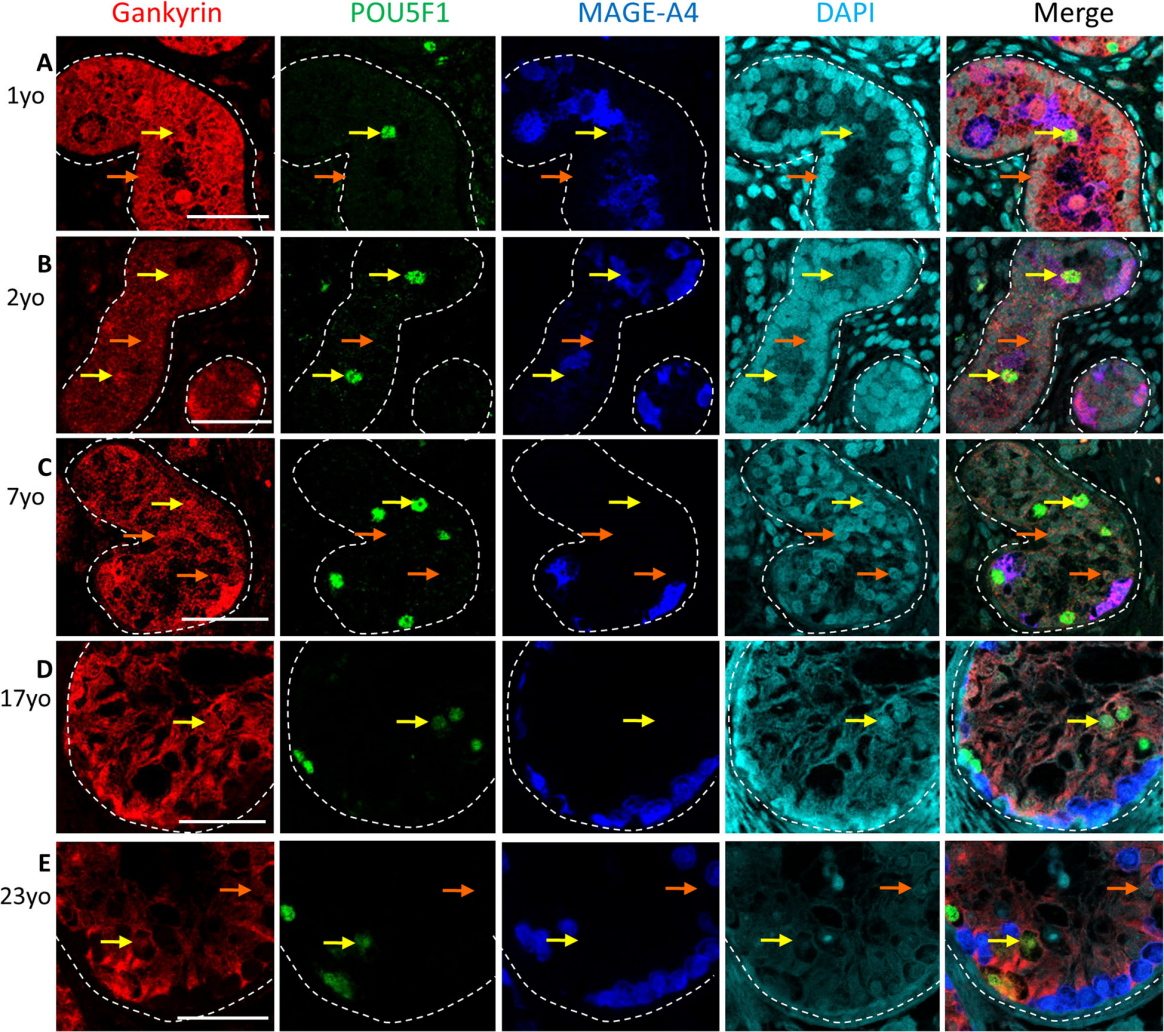
Gankyrin expression pre-invasive germ cells. **a**) 1 year old - maturation delay; **b**) 2 year old – maturation delay; **c**) 7 year old pre-invasive TGCC; **d**)17 year old pre-invasive TGCC; and **e**) 23 year old pre-invasive TGCC. Yellow arrows – GCNIS cells, orange arrows – Sertoli cells. This experiment was performed along with human fetal testis samples, no primary antibody control is the same as on Fig. 1, Scalebars - 50 μm.

**Fig. 3:**
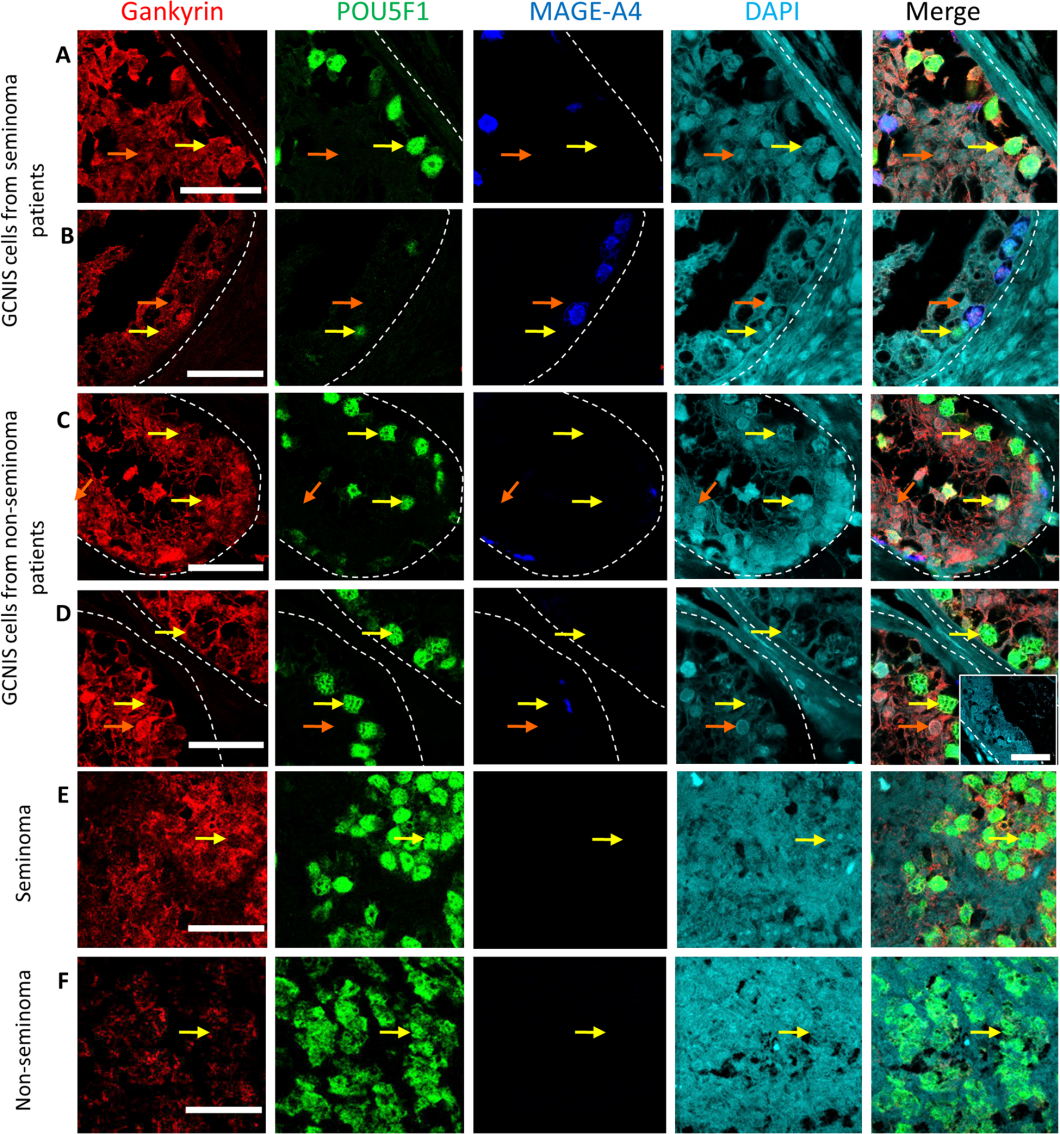
Gankyrin expression in invasive TGCC. **a**-**d** Gankyrin (red), POU5F1 (green; GCNIS cells), and MAGE-A4 (blue; spermatogonia) expression in GCNIS containing tubules from patients with invasive TGCC, yellow arrows – GCNIS cells, orange arrows – Sertoli cells. **e** and **f** Gankyrin (red), POU5F1 (green; seminoma cells), and MAGE-A4 (blue) expression in seminoma cells (**e**) and a mixed TGCC (**e**), inset on D – no primary antibody control. Scalebars - 50 μm.

According to TGCC pathogenesis, there is a block of differentiation of gonocytes, which then become GCNIS and remain in the testis. After puberty these cells give rise to tumours. The gonocytes are the precursor cells of GCNIS and therefore the expression profile of gonocytes compared to GCNIS is important to examine potential mechanism of malignant transformation. In human fetal testis, gonocytes did not express nuclear Gankyrin whereas in samples with maturation delay and in GCNIS from both pre-pubertal and adult patients with pre-invasive TGCC, nuclear Gankyrin was observed (yellow arrows, Fig. 2). Similarly, nuclear Gankyrin expression was observed in a subset of GCNIS cells from adult patients with invasive TGCC (yellow arrows, Fig. 3a-d). In order to examine whether there was a difference in nuclear Gankyrin expression between MAGE-A4^+^ and MAGE-A4^−^ GCNIS cells in GCNIS from patients with pre-invasive (*n* = 6) and invasive (n = 6) TGCC, we quantified Gankyrin expression in each sub-population. An increased proportion of nuclear Gankyrin expression was observed in POU5F1^+^/MAGE-A4^−^ GCNIS compared to POU5F1^+^/MAGE-A4^+^ GCNIS (Fig. S1). Gankyrin was also detected in the cytoplasm of seminoma tumour cells and the seminoma component of mixed non-seminoma, which was determined by POU5F1 expression (Fig. 3e and f).

### Knock-down of Gankyrin expression in NTera2 cells results in a reduction in cell number without effects on POU5F1 expression

**Fig. 4:**
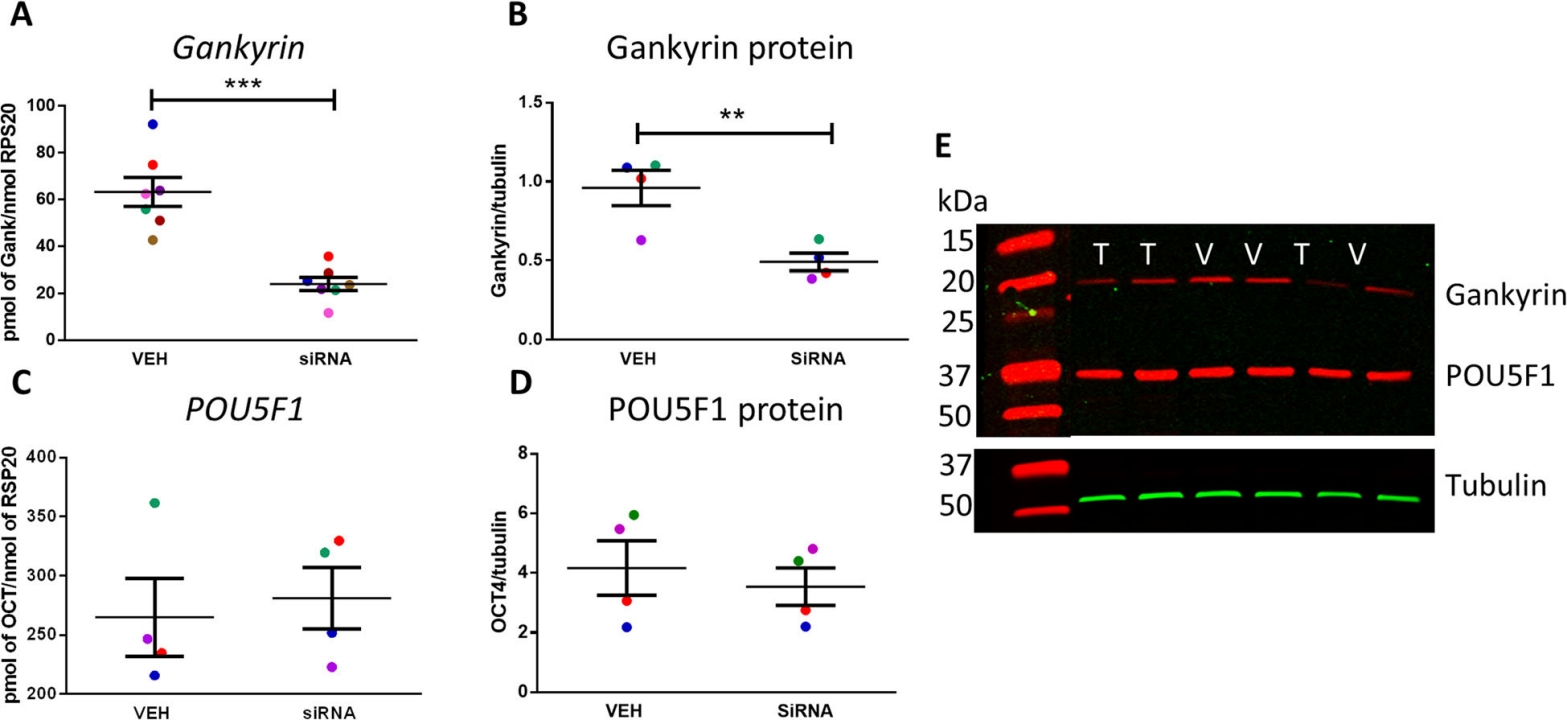
Effects of Gankyrin knock-down on Gankyrin and POU5F1 expression. Relative Gankyrin mRNA(**a**) and protein (**b**) expression in NTera2 cells after Gankyrin knock-down. Relative POU5F1 mRNA (**c**) and protein (**d**) expression in NTera2 cells after Gankyrin knock-down. **e** Representative Western blot for Gankyrin and POU5F1 expression in (V) vehicle and Gankyrin siRNA (T) transfected NTera2 cells. Tubulin was used as a loading control. Data analysed by paired *t*-test ± SEM, ***p < 0.001, **p < 0.01. Each data point represents the mean of an individualexperiment, each with 3 replicates. Paired samples from an individual experiment are represented by the same colour.

To investigate the role of Gankyrin in malignant germ cells, we used the embryonal carcinoma cell line (NTera2) to perform Gankyrin knock-down in vitro using an siRNA approach. Gankyrin mRNA expression was significantly reduced (62%; *p* < 0.001) after 24 h, with a similar reduction (50%; *p* < 0.01) at the protein level (Fig. 4a and b). The reduction in Gankyrin expression did not affect POU5F1 expression at either the mRNA or protein level (Fig. 4c-e).

**Fig. 5:**
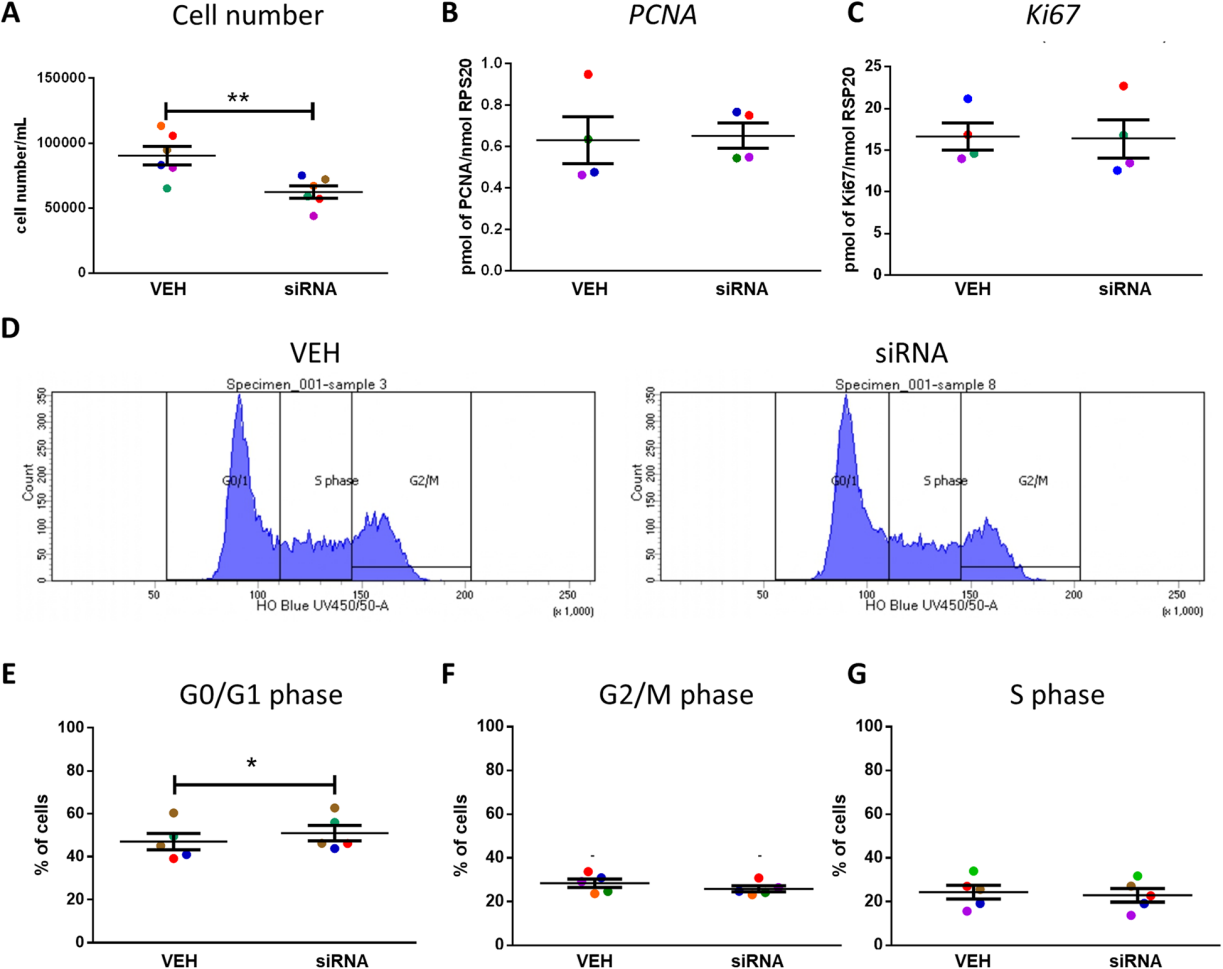
Effect of Gankyrin knock-down on cell number, cell proliferation and cell cycle of NTera2 cells. **a** Gankyrin knock-down effect on NTera2 cell number. Relative *PCNA* (**b**) and *ki67* (**c**) expression after 24 h of Gankyrin siRNA transfection. Distribution of NTera2 cells in GO/G1 phase (**d**), G2/M phase (**e**) or S phase (**f**). Data analysed by paired *t*-test, means ± SEM, ** *p* < 0.001, *p < 0.05. Each data point represents the mean of an individual experiment, each with an 3 replicates. Paired samples from an individual experiment are represented by the same colour. (**g**) Representative image of the flow cytometry plot obtained during cell cycle experiments.

Knock-down of Gankyrin expression resulted in a significant reduction in the number of NTera2 cells (32%; p < 0.01, Fig. 5a). To investigate this further, we determined the expression of genes involved in cell proliferation. Gankyrin knock-down did not affect the expression of either *PCNA* or *Ki67* (Fig. 5b and c); however, cell cycle analysis demonstrated a small but significant increase (4%; *p* < 0.05) in the proportion of NTera2 cell in G0/G1 phase, with no significant effects on other phases of the cell cycle (Fig. 5d-g).

**Fig. 6:**
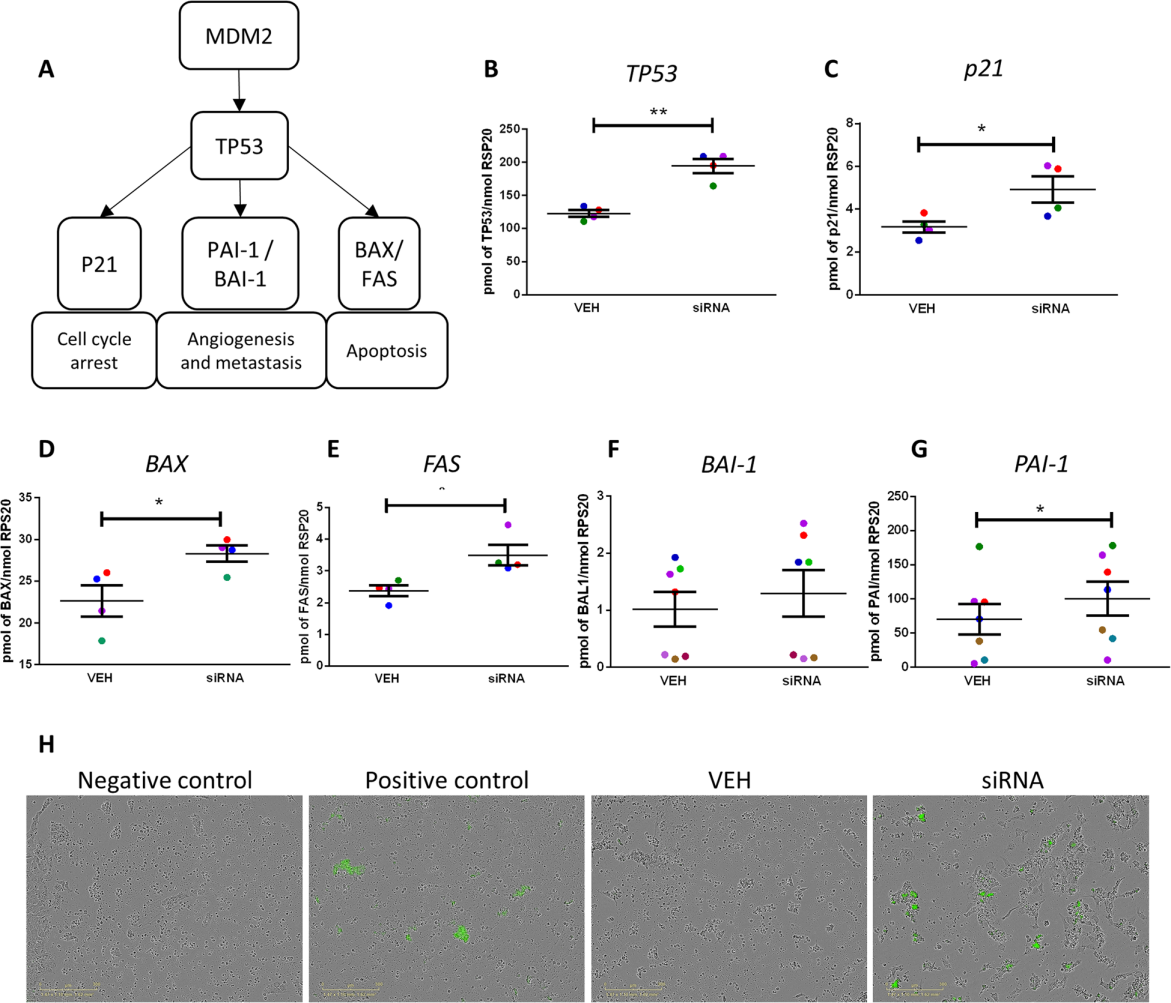
Gankyrin knock-down effect on TP53 and apoptosis. **a** TP53 pathway showing genes of interest in this study. Relative (**b**) *TP53* (*n* = 4), (**c**) *P21* (*n* = 4), (**d**) *BAX* (*n* = 4), (**e**) *FAS* (*n* = 4), (**f**) *BAI-1* (*n* = 6), (**g**) *PAI-1* (*n* = 7) mRNA expression in NTera2 cells after Gankyrin knock-down. Data (**b**-**g**) analysed by paired *t*-test, means ±SEM, *p < 0.05, **; *p* < 0.001. Each data point represents the mean of an individual experiment, each with 3 replicates. Paired samples from an individual experiment are represented by the same colour. H) Effects of Gankyrin knock-down on apoptosis in NT2 cells using Nucview apoptosis (CC3) probe. Representative images of four separate experiments, each with 3 replicates.

To determine whether the effects on cell number involved activation of apoptosis and/or cell cycle arrest, we investigated the TP53 pathway (Fig. 6, A). Knock-down of Gankyrin expression resulted in a significant increase in *TP53* expression (Fig. 6b) and several downstream genes including *P21*, *BAX*, *FAS*, and *PAI-I* (Fig. 6c-g). Functional image-based analysis using a cell-permeable fluorescent caspase biosensor revealed knockdown of Gankyrin resulted in activation of Cleaved Caspase 3 (CC3) mediated apoptosis, whilst no apoptotic cells were identified in controls (Fig. 6h).

### Gankyrin knock-down enhances cisplatin mediated cell death in NTera2 cell

**Fig. 7:**
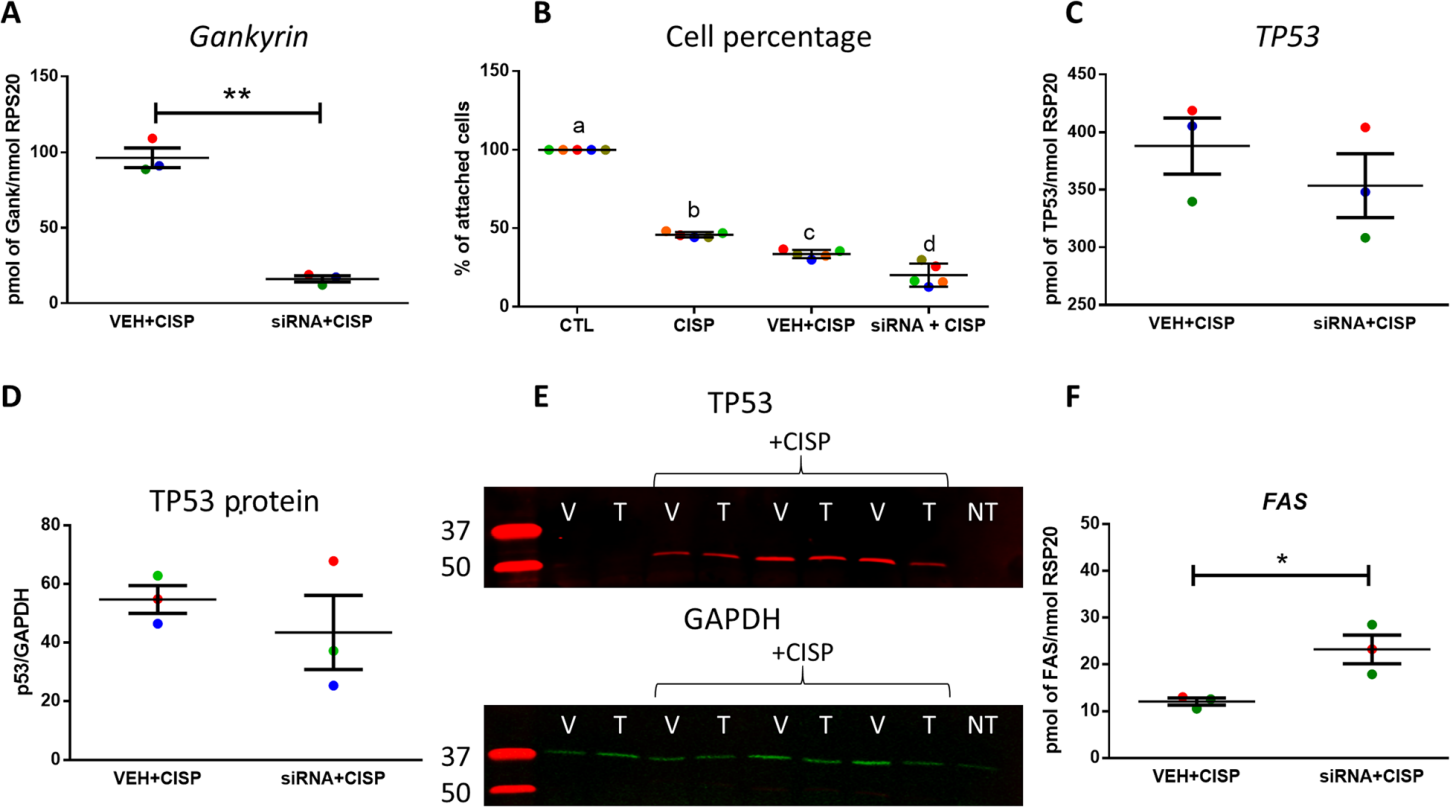
Effect of Gankyrin knock-down on cisplatin sensitivity in NTera2 cells. **a** Gankyrin mRNA expression after Gankyrin knock-down in cisplatin (20 nM) exposed NTera2 cells. **b** Gankyrin knock-down and cisplatin treatment effect on the percentage of surviving cells Gankyrin knock-down and cisplatin treatment effects on (**c**) *TP53* mRNA and (**d**) protein expression. **e** Representative image for TP53 western blot in Vehicle (V) and Gankyrin siRNA transfected (T) samples with and without cisplatin treatment and a no treatment control (NT). **f** Relative *FAS* mRNA expression after Gankyrin knock-down and cisplatin treatment. CTL: control, CISP: cisplatin, VEH + CISP: vehicle and cisplatin, siRNA+CISP: Gankyrin siRNA+cisplatin. Data analysed by paired *t*-test, means ±SEM, **p < 0.001, *p < 0.05. Each data point represents the mean of an individual experiment, each with 3 replicates. Paired samples from an individual experiment are represented by the same colour.

*TP53* has been shown to be important in mediating the cytotoxic effect of cisplatin in TGCC [33, 43, 44], therefore we investigated the role of Gankyrin in cisplatin sensitivity in NTera2 cells. We confirmed the siRNA mediated knock-down of Gankyrin expression in cisplatin exposed NTera2 cells (Fig. 7a), and found that this resulted in a significant reduction in the percentage of recovered live cells compared to non-transfected untreated controls (80%, *p* < 0.05) and non-transfected cisplatin treated controls (50%, p < 0.05) (Fig. 7b). There was no effect of Gankyrin knock-down on TP53 mRNA or protein expression (Fig. 7c-e) however there was a significant increase in *FAS* mRNA expression in cisplatin transfected cells (Fig. 7f).

## Discussion

The present study represents the first description of Gankyrin expression in the normal human testis, testis with maturation delay and in GCNIS from patients with pre-invasive and invasive TGCC. In the human fetal testis, we have shown that whilst nuclear expression of Gankyrin was not detected in gonocytes (POU5F1^+^), nuclear Gankyrin was observed in the nuclei of a sub-set of (pre) spermatogonia (MAGE-A4^+^). This suggest Gankyrin might be involved in normal germ cell differentiation. GCNIS is believed to result from failure of differentiation from gonocyte (POU5F1^+^) to (pre) spermatogonia (POU5F1^−^) [4, 5]. Our observation that nuclear Gankyrin is not expressed in gonocytes (POU5F1^+^/Gankyrin^−^) but expressed in gonocytes from samples with maturation delay and pre-GCNIS (POU5F1^+^/Gankyrin^+^) indicates that Gankyrin expression is associated with the early stage of TGCC development. Furthermore, within the GCNIS cell populations, Gankyrin expression is present in a higher proportion of POU5F1^+^/MAGE-A4^−^ compared with POU5F1^+^/MAGE-A4^+^cells, which may reflect an increased oncogenic potential in the in the more proliferative POU5F1^+^/MAGE-A4^−^ population [45].

Previous studies have demonstrated that Gankyrin prevents POU5F1 degradation in HCC [23] and loss of Gankyrin can reduce the oncogenic potential of tumour cells through interaction with MAGE-A4 [26]. This, combined with our previous finding of reduced oncogenic potential in the POU5F1^+^/MAGE-A4^+^ population of GCNIS cells compared with POU5F1^+^/MAGE-A4^−^ population [14] led us to hypothesize that Gankyrin expression might play a similar role in the pathogenesis of TGCC by preventing POU5F1 degradation and contributing to malignant progression.

Therefore, we investigated the effects of Gankyrin knock-down in NTera2 cells, an established embryonal carcinoma cell line which is widely used in studies relating to TGCC [6, 46–48]. Transfection with siRNA targeting Gankyrin resulted in a significant reduction (62%; *p* < 0.001) in *Gankyrin* expression. Gankyrin knock-down did not affect POU5F1 mRNA or protein expression in NTera2 cells demonstrating that Gankyrin does not prevent POU5F1 degradation in this cell line. Interestingly, we did find that Gankyrin knock-down led to a significant reduction in cell number suggesting a possible role for this protein in the survival of malignant germ cells. Several studies have demonstrated effect of Gankyrin on oncogenic potential in hepatocellular carcinoma cells due to increased cell proliferation and malignant transformation of normal hepatocytes [20, 23, 24, 49, 50].

Given that knock-down of Gankyrin expression did not affect the mRNA expression levels of proliferation markers and induced only minor changes in the proportion of cells in the different phases of cell cycle, we speculated that the reduction in cell number may be as a result of an increase in apoptosis. A number of pro-apoptotic genes are located downstream of *TP53* and we found that *TP53* expression is upregulated following knock-down of Gankyrin in NTera2 cells, which is in keeping with the results of a previous study [36]. Furthermore, we have demonstrated that Gankyrin knock-down results in an increased expression of apoptosis genes *BAX* and *FAS*, both of which are downstream of *TP53*. Down-regulation of Gankyrin also induces apoptosis in hepatocellular carcinoma cells with wild type TP53 [35] whilst increased expression of Gankyrin inhibits apoptosis by causing degradation of *TP53* protein and reduced transcription of its downstream apoptotic genes [35]. Furthermore, apoptotis was induced following Gankyrin down-regulation, as indicated by Cleaved Caspase 3 activity. Taken together these results suggest that following Gankyrin knock-down in NTera2 cells the reduction in cell number is likely to be mediated by an increase in apoptosis mediated through the TP53 signalling pathway leading to increased expression of the apoptotic genes *BAX* and *FAS*.

Cisplatin, is the chemotherapeutic drug of choice for treatment of TGCC, acting through the *TP53* pathway to induce DNA damage [33]. The expression of wildtype *TP53* in TGCC has been proposed to be a key determinant for the effectiveness of cisplatin treatment [30]. This might be related to the expression of a selected number of embryonic microRNAs [51]. Previous studies have reported that *TP53* mutations did not occur in TGCC [52], however recent studies have shown that 10 out of 148 patients with seminoma (7%) have a *TP53* mutation [53]. Although *TP53* is abundantly present in its wildtype form in TGCC, it has also been suggested that *TP53* is inactive in TGCC, given that its downstream genes have been indicated as non-detectable [30]. Recent studies have demonstrated that knockdown of TP53 in NTera2 cells resulted in reduced cisplatin mediated apoptosis [33, 34]. Therefore, given that we identified an effect of Gankyrin knock-down on the TP53 and BAX/FAS apoptosis pathway, we speculated that manipulation of Gankyrin might modulate the effect of cisplatin in TGCC. To test this, we combined Gankyrin knock-down with cisplatin treatment in NTera2 cells. We showed that Gankyrin knock-down enhances the reduction in cell number caused by cisplatin treatment by 13% (*p* < 0.05), compared to cisplatin treatment alone. Taken together, these results suggest that Gankyrin plays a role in cisplatin sensitivity and resistance.

## Conclusion

In conclusion, we have demonstrated that Gankyrin is expressed in the sub-populations of germ cells in the normal fetal and adult testis, as well as in pre-invasive and invasive TGCC tissue from patients. We have also demonstrated that repression of Gankyrin in NTera2 cells results in a reduction in total cell number and enhances the cytotoxic effect of cisplatin in this TGCC cell line. Together our results suggest that Gankyrin may be a potential target for treatment of TGCC either as an adjunct to cisplatin or in cisplatin resistant tumours.

## Supplementary information

**Additional file 1 Figure S1.** Nuclear Gankyrin expression in POU5F1^+^/MAGE-A4^−^ GCNIS cells and in POU5F1^+^/MAGE-A4^+^ GCNIS cells in samples from pre-invasive GCNIS and invasive TGCC patients (*n* = 12). Data analysed by paired *t*-test, means ± SEM.

## Data Availability

The data to support the findings of this study are available upon reasonable request from the corresponding author, but restrictions apply to the availability to these data, which were used under license for the current study, and so are not publicly available.

## Abbreviations

DSD: Disorders of Sex Development
GCNIS: Germ Cell Neoplasia in situ
HCC: Hepatocelular Carcinoma
NChS: Normal Chicken Serum
RIPA: Radioimunoprecipitation Assay
TBS: Tris Buffer Saline
TGCC: Testicular Germ Cell Cancer
